# Bidirectional modulation of somatostatin-expressing interneurons in the basolateral amygdala reduces neuropathic pain perception in mice

**DOI:** 10.3389/fpain.2025.1602036

**Published:** 2025-08-13

**Authors:** Aditya Apte, Julia Fernald, Cody Slater, Marc Sorrentino, Brett Youngerman, Qi Wang

**Affiliations:** ^1^ Department of Biomedical Engineering, School of Engineering and Applied Science, Columbia University, New York, NY, United States; ^2^ Vagelos College of Physicians and Surgeons, Columbia University, New York, NY, United States; ^3^ Department of Neurosurgery, Columbia University Irving Medical Center, Columbia University, New York, NY, United States

**Keywords:** basolateral amygdala, neuropathic pain, somatostatin, parvalbumin, chemogenetic manipulation, von Frey test, Hargreaves test, acetone test

## Abstract

**Introduction:**

Neuropathic pain is characterized by mechanical allodynia and thermal (heat and cold) hypersensitivity, yet the underlying neural mechanisms remain poorly understood.

**Methods:**

Using chemogenetic excitation and inhibition, we examined the role of inhibitory interneurons in the basolateral amygdala (BLA) in modulating pain perception following nerve injury.

**Results:**

Chemogenetic excitation of parvalbumin-positive (PV^+^) interneurons significantly alleviated mechanical allodynia but had minimal effects on thermal hypersensitivity. However, inhibition of PV^+^ interneurons did not produce significant changes in pain sensitivity, suggesting that reductions in perisomatic inhibition do not contribute to chronic pain states. In contrast, bidirectional modulation of somatostatin-positive (SST^+^) interneurons influenced pain perception in a modality-specific manner. Both excitation and inhibition of SST^+^ interneurons alleviated mechanical allodynia, indicating a potential compensatory role in nociceptive processing. Additionally, SST^+^ neuron excitation reduced cold hypersensitivity without affecting heat hypersensitivity, whereas inhibition improved heat hypersensitivity but not cold responses.

**Discussion:**

Our findings suggest that, in addition to PV^+^ neurons, SST^+^ interneurons in the BLA play complex roles in modulating neuropathic pain following nerve injury and may serve as a potential target for future neuromodulation interventions in chronic pain management.

## Introduction

Neuropathic pain is a chronic and debilitating condition that arises from damage to the nervous system, often manifesting in the absence of noxious stimuli. This form of pain is typically associated with disorders such as diabetes, trauma, or neurodegenerative diseases, and is characterized by spontaneous bouts of pain, allodynia, and hyperalgesia. The global prevalence of neuropathic pain is estimated at approximately 8% of the population and has a substantial impact on quality of life, making it a significant public health issue (1, 2). In addition to its negative impact on the quality of life, recent studies have suggested that severe chronic pain is strongly correlated with the rate of cognitive decline and an elevated risk of dementia (3). Despite the burden of neuropathic pain on society, many current pharmacological approaches fail to provide sustained relief without undesirable side effects (4). Consequently, there is a critical need for better therapeutic interventions that target the underlying neural mechanisms of pain perception (5).

Perceptual processing is a highly adaptive process that depends on the characteristics of sensory inputs and behavioral state (6–10). Chronic pain is increasingly recognized as a disorder of maladaptive neural plasticity, involving long-term changes in synaptic strength, inhibitory tone, and neuromodulatory systems (11, 12). The basolateral amygdala (BLA) is a key structure in this process, integrating sensory input with emotional valence and contributing to the persistence of pain states (13–15). Lesion studies and electrophysiological recordings have demonstrated that neuronal activity within the BLA correlates with both the intensity and affective dimensions of pain (16), with evidence suggesting that synaptic and cellular reorganization within this region facilitates the transition from acute to chronic pain (17).

A growing body of work suggests that inhibitory interneurons in the BLA play a pivotal role in regulating pain-related plasticity. Parvalbumin-expressing (PV^+^) interneurons provide perisomatic inhibition that stabilizes excitatory network activity and contributes to pain modulation (18). Conversely, somatostatin-expressing (SST^+^) interneurons primarily target distal dendrites, influencing synaptic integration and network dynamics (19, 20). While PV^+^ and SST^+^ interneurons are well-characterized in cortical circuits, their functional contributions to BLA-dependent pain modulation remain not fully understood.

Emerging research shows that these interneuron populations exhibit anatomical and functional heterogeneity. Previous studies have identified multiple subgroups of SST^+^ interneurons in the central nucleus of the amygdala and BLA, some of which project to downstream autonomic and nociceptive processing centers (21–26). Similarly, PV^+^ interneurons in the BLA have been implicated in the regulation of oscillatory network activity and sensory gating (27, 28). Given the involvement of these interneurons in regulating emotional and sensory processing, it is plausible that their dysfunction contributes to chronic pain pathology.

In this study, we investigated the specific contributions of PV^+^ and SST^+^ interneurons in regulating nociceptive processing in the BLA. Using a chemogenetic approach, we examined how manipulation of these interneuron subtypes affects mechanical, thermal, and cold allodynia. Our findings suggest that SST^+^ and PV^+^ neurons exert distinct yet complementary roles in shaping pain-related behaviors, with potential implications for targeted neuromodulation therapies.

## Methods

### Animals and surgical procedures

All experimental procedures were approved by the Columbia University Institutional Animal Care and Use Committee (IACUC) and were conducted with compliance with NIH guidelines. Twenty-four adult mice of both male and female sex (12 female and 12 male), aged 3–6 months, were used in the experiments. The strains used were SST-IRES-Cre (RRID: IMSR_JAX:013044, 7 females and 8 males in which 3 females and 3 males were used for BLA SST+ neuron excitation experiments and 4 females and 5 males were used for BLA SST+ neuron inhibition experiments.) and PV-IRES-Cre (RRID: IMSR_JAX:017320, 5 females and 4 males in which 2 females and 2 males were used for BLA PV+ neuron excitation experiments and 3 females and 2 males were used for BLA PV+ neuron inhibition experiments). All mice were kept under a 12-hour light-dark cycle.

#### Intracranial adeno-associated virus injection

The procedure for injecting the viral vector solutions is similar to that in our previous studies (29). Mice were initially anesthetized with 5% isoflurane in an induction chamber. Once the animal's condition stabilized, it was mounted onto a stereotaxic frame using a pair of non-puncture ear bars (Kopf Instruments, Tujunga, CA). The head was carefully leveled. Throughout the surgical procedure, 2% isoflurane was used to maintain the surgical plane of anesthesia, and the animal's body temperature was maintained at 38°C using a feedback-controlled heating pad (FHC, Bowdoinham, ME). Buprenorphine (0.05 mg/kg, subcutaneous) was subsequently administered to provide preemptive analgesia.

During aseptic preparation, the fur on the skull was shaved to create a clean site for incision. The area was sterilized using three alternating passes of 70% alcohol swabs and betadine, followed by a subcutaneous injection of 2% lidocaine into the scalp. An incision was then made to expose the skull, and a burr hole was drilled above the right BLA (AP: −1.4 mm, ML: 2.8 mm, DV: −4.9 mm) (30), with saline applied to the craniotomy to prevent drying of the brain surface. We targeted the right BLA based on evidence from earlier research that linked tactile hypersensitivity to increased activity in the amygdala on the right side of the mouse's brain (16, 31–33). Pulled capillary glass micropipettes were back-filled with 150 nl AAV solution (pAAV1-hSyn-DIO-hM3D(Gq)-mCherry or pAAV1-hSyn-DIO-hM4D(Gi)-mCherry), which was subsequently injected into the BLA at 0.7 nl/s using a precision injection system (Nanoliter 2020, World Precision Instruments, Sarasota, FL). The pipette was left in place for at least 10 min following injection and slowly withdrawn. Following the withdrawal of the pipette, skin was closed with Vetbond. Baytril (5 mg/kg) and Ketoprofen (5 mg/kg) were administered postoperatively for 5 days.

#### Constriction nerve injury (CNI) procedure

Nerve injury surgeries were performed 10 days following AAV injection surgery. The injury was induced using a chronic constriction method similar to previously described (34). In aseptic surgeries, mice were initially anesthetized with isoflurane. To access the left lateral thigh of the mouse, the animal was placed on its side, and limbs were secured to the surgical surface to ensure stability of the leg during surgery. A 1 cm thick gauze pad was placed between the hind legs to provide support to the leg and make access more feasible. After shaving the surgical site, a 1 cm long incision was made in the proximal one third of the lateral thigh of the left leg. The sciatic nerve was exposed by opening muscle fascia between the gluteus superficialis and biceps femoris muscles using sterilized toothpicks to push apart muscle fibers without causing damage to nearby blood vessels. The sciatic nerve was then raised from the cavity using fine-tip forceps inserted under the nerve and gently stretched using said forceps. Three nylon ligatures were tied around the sciatic nerve 2 mm apart, to cause enough constriction to the nerve without preventing epineural blood flow. The wound was closed with absorbable sutures in the muscle and skin. Baytril (5 mg/kg) was administered subcutaneously and Triple antibiotics ointment was applied for 3 days in post-operative care. The animal is allowed to recover from surgery for 3 days before injured baseline behavioral testing begins.

### Chemogenetic manipulation

To test the role of SST+ or PV+ interneurons in the BLA in pain behavior, we performed 7 days of chemogenetic manipulation (i.e., treatment) and 3 days of saline control (sham-treatment). For all chemogenetic manipulation experiments, clozapine-N-oxide (CNO; Hello Bio, NJ) was injected i.p. (3 mg/kg body weight) 30 min prior to testing. On sham-treatment days, an equivalent volume of saline was injected i.p. 30 min prior to testing. Treatment and sham-treatment sessions were randomly interleaved and were blind to the experimenter who conducted behavioral tests.

### Nociceptive behavioral assays

**Figure 1:**
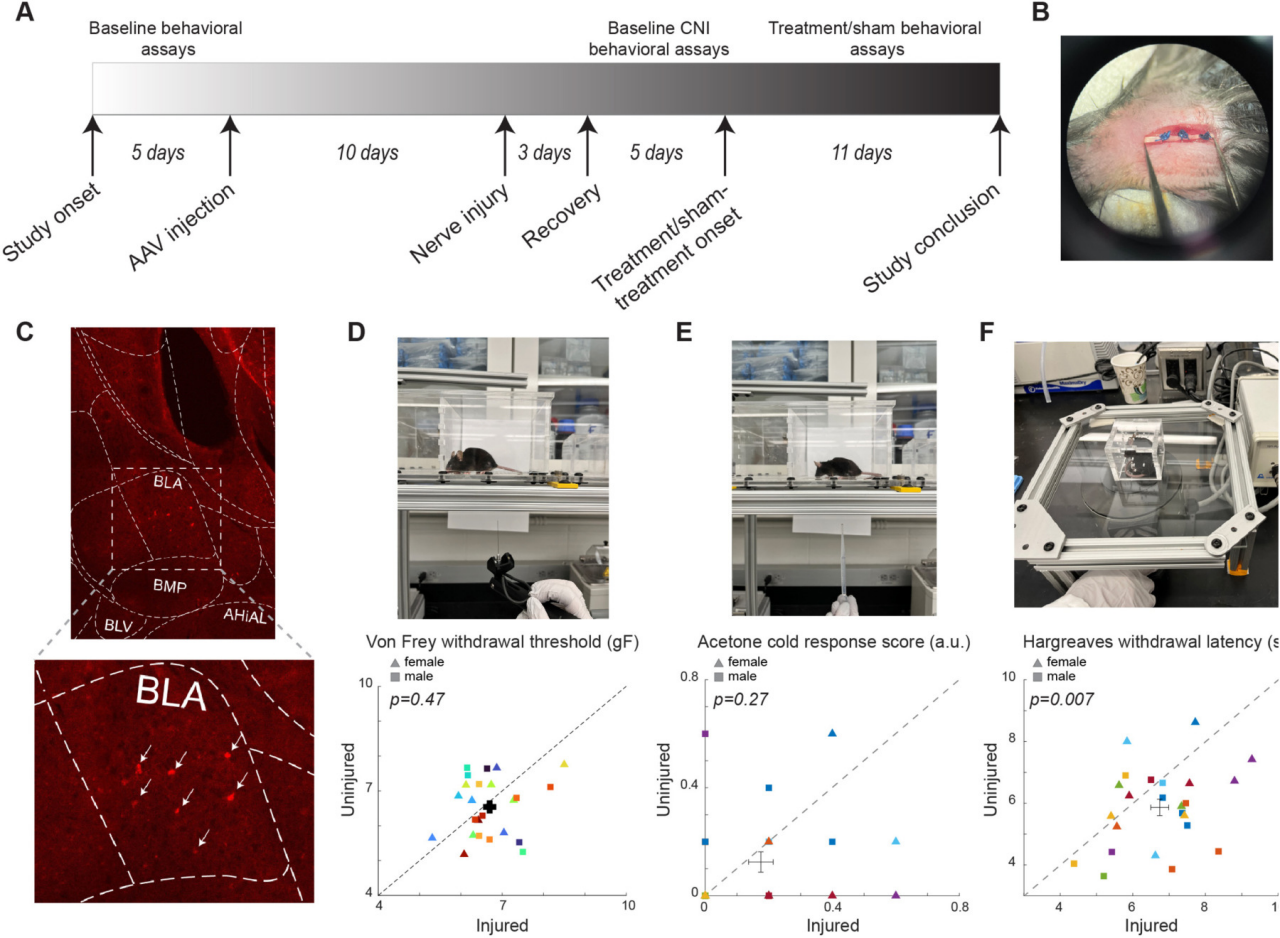
Experimental setup and baseline results of the behavioral assays. **(A)** The timeline of the study. **(B)** Photo of example CNI. **(C)** Example immunohistology confirming expression of DREADD receptors in SST+ neurons in the BLA. White arrows indicate SST+ neurons. **(D)** Photo of a von Frey test (top) and withdrawal thresholds in von Frey tests prior to CNI (bottom). 106 sessions from 12 female (5 PV-Cre, and 7 SST-Cre) and 12 male (4 PV-Cre, and 8 SST-Cre) mice. **(E)** Photo of an acetone test (top) and response scores in acetone tests prior to CNI (bottom). 24 sessions from 12 female (5 PV-Cre, and 7 SST-Cre) and 12 male (4 PV-Cre, and 8 SST-Cre) animals. **(F)** Photo of a Hargreaves test (top) and withdrawal latencies in Hargreaves tests prior to CNI (bottom). 24 sessions from 12 female (5 PV-Cre, and 7 SST-Cre) and 12 male (4 PV-Cre, and 8 SST-Cre) animals. Triangle symbols denote female animals while square symbols denote male animals. Each symbol represents an individual animal. Error bars indicate 1 S.E.M.

All behavioral assays were performed by an experimenter blinded to the mouse group and administered treatment. Baseline data were collected within the 5 days preceding the AAV injection surgery. CNI baseline measurements were obtained between days 4 and 8 following the CNI procedure. The effects of BLA interneuron manipulations were assessed within 11 days after the completion of CNI baseline data collection (Figure 1A).

#### Von Frey tests for mechanical allodynia

Mice were habituated on an elevated acrylic mesh platform in 4 × 4 × 3 inch acrylic boxes for 30 min before mechanical stimulation with a von Frey filament (Figure 1D) (35). The sensitivity of the paw to mechanical stimulation was measured using an electronic von Frey aesthesiometer (eVF, Model: 38450, Ugo Basile). Once the filament is applied, the eVF records the force readout (gF) and latency (s). Mechanical paw withdrawal threshold was defined as the force applied by the filament causing the paw to withdraw. Three measurements were taken for each of the hindpaws, ipsilateral and contralateral to the injury side, per session. Each animal underwent 3–5 baseline sessions prior to CNI, 3–5 sessions to establish the CNI baseline, and 11 sessions to assess the effects of BLA manipulations (including 9 treatment and 2 sham sessions for one mouse, and 8 treatment and 3 sham sessions for the remaining 23 mice).

#### Acetone test for cold sensitivity

Mice were habituated on an elevated acrylic mesh platform in 4 × 4 × 3 in acrylic boxes for 30 min prior to application of acetone droplet (Figure 1E). Acetone was loaded into a 5 ml pipette and a drop was lightly applied through the acrylic mesh to the plantar surface of the hindpaw, without touching the paw with the pipette to avoid false response (35). Nociceptive responses after applying acetone droplet to the mouse's hindpaw were quantified using a version of the scoring system described (36) where 0 = minimal to no lifting, licking, or shaking of the hindpaw; 1 = lifting, licking, and/or shaking of the hindpaw, continuing for between 1 and 5 s; 2 = lifting, licking, and/or shaking of the hindpaw, prolonged or repetitive, extending beyond 5 s after application of acetone (36). Responses to acetone were gauged 5 times per hindpaw per session. Each animal underwent 1 session before CNI, 1–2 sessions to establish the CNI baseline, and 3 sessions (2 treatment and 1 sham) for evaluating the effects of BLA manipulations.

#### Hargreaves tests for heat sensitivity

Mice were habituated on an elevated ⅛ inch thick glass platform in a 2 × 2 × 2 inch ventilated acrylic box for 30 min prior to application of a thermal stimulus using a constant radiant heat source (Model: 7371, Ugo Basile) with an active intensity of 25% to the plantar surface of each hind paw through the glass surface (Figure 1F) (35). The latency for paw withdrawal was measured five times per hindpaw. Each animal underwent 1 session before CNI, 1 session to establish the CNI baseline, and 3 sessions (2 treatment and 1 sham) to assess the effect of BLA manipulations.

### Histology

At the end of the study, a subset of SST-Cre mice from the experimental cohort was anesthetized and transcardially perfused with phosphate buffered saline (PBS) followed immediately by ice-cold 4% paraformaldehyde (PFA). The brain was carefully extracted from the skull and post-fixed for 24 h at 4°C in 4% PFA, and then preserved in a 30% sucrose (wt/vol) PBS solution for 72 h at 4 °C. After 3 days, extracted brains were embedded in optimum cutting temperature compound, and 30-μm coronal slices were obtained using a cryostat (CM1950, Leica Microsystems), and placed on a glass microscope slide. Brain slices were washed three times in PBS followed by placement and mounting of a coverslip using Fluoromount-G medium with DAPI. The slices were imaged using 20x under a confocal microscope (Nikon Ti2) with a spinning disk (Yokogawa CSU-W1). Since we used SST-Cre and PV-Cre transgenic mouse lines in combination with AAV vectors encoding a double-floxed inverted DREADD-mCherry sequence for Cre-dependent expression, DREADD expression in SST+/PV+ neurons was confirmed by detecting unamplified mCherry fluorescence signals.

### Data analysis

All data analyses were conducted on individual animals. The averages and standard errors of means were then calculated across animals for each experimental group. For von Frey and Hargreaves tests, the normalized change between two hindpaws was calculated as $V_{\mathrm{uninjured}}-V_{\mathrm{injured}}/V_{\mathrm{uninjured}}+V_{\mathrm{injured}}$, where $V_{\mathrm{uninjured}}$ is the measurement from the hindpaw on the uninjured side, while $V_{\mathrm{injured}}$ is the measurement from the hindpaw on the injured side. Because in acetone tests, the hindpaw on the injured side usually had a higher score, the normalized change was calculated as $V_{\mathrm{injured}}-V_{\mathrm{uninjured}}/V_{\mathrm{injured}}+V_{\mathrm{uninjured}}$, where $V_{\mathrm{uninjured}}$ is the measurement from the hindpaw on the uninjured side, while $V_{\mathrm{injured}}$ is the measurement from the hindpaw on the injured side. Following normalization, undefined values were excluded from the data analysis.

### Statistics

One-sample Kolmogorov–Smirnov test was used to verify the normality of the data. For data with a normal distribution, a Student's *t*-test was performed. Otherwise, the Mann–Whitney *U*-test for unpaired samples or the Wilcoxon Signed Rank test was used for paired samples. One-way ANOVA was performed to determine significance of normalized plots comparing injured baseline, activation/inhibition, and saline-control responses.

## Results

**Figure 2:**
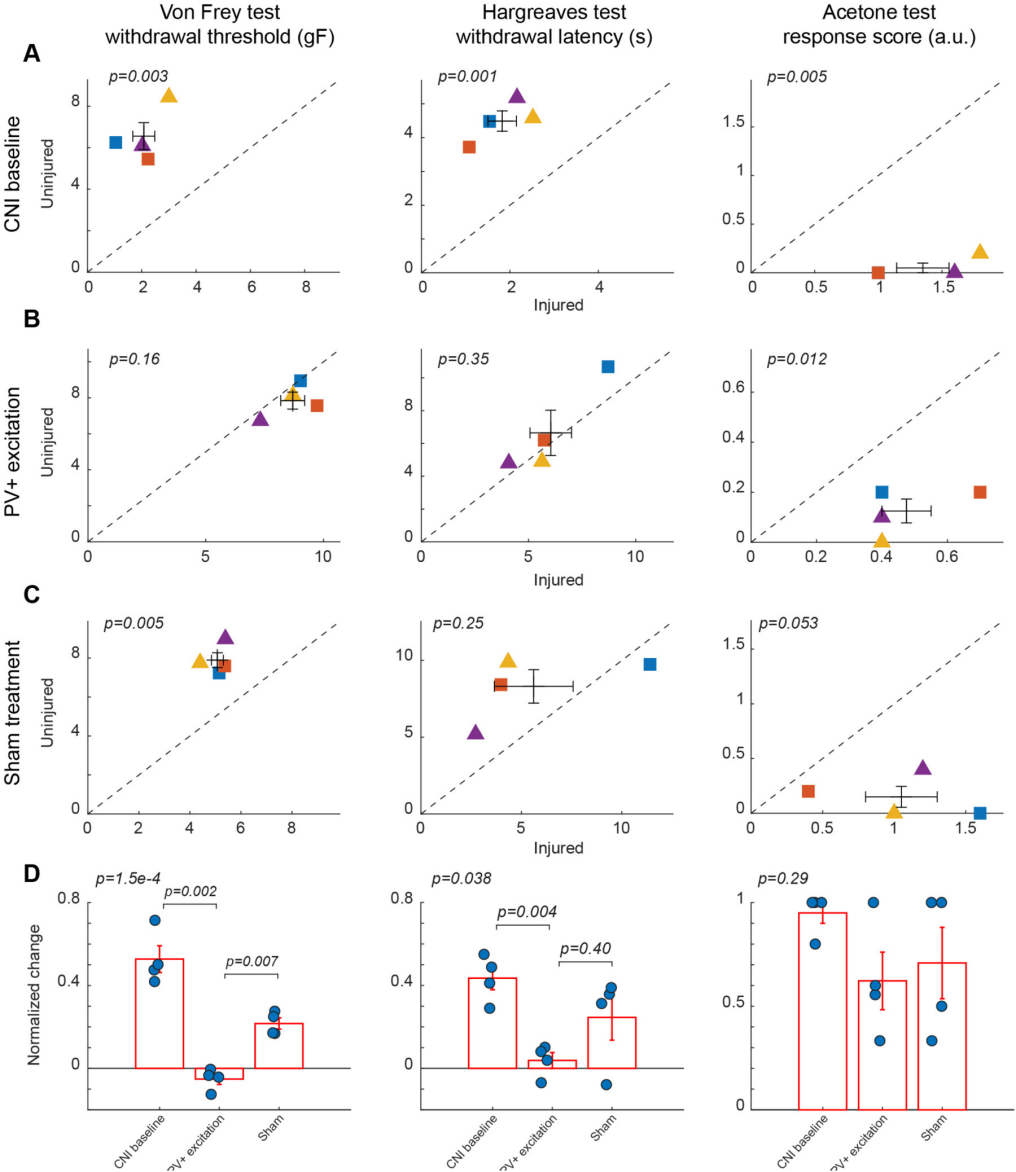
Effects of activation of PV+ interneuron in the BLA. **(A)** Behavioral outcomes in the von Frey, Hargreaves, and acetone tests after CNI. Von Frey data are from 15 sessions from 2 female and 2 male PV-Cre mice. Hargreaves data are from 4 sessions from 2 female and 2 male PV-Cre mice. Acetone data are from 4 sessions from 2 female and 2 male PV-Cre mice. **(B)** Behavioral outcomes in the von Frey, Hargreaves, and acetone tests after CNI with activation of PV+ neurons in the BLA. Von Frey data are from 33 sessions from 2 female and 2 male PV-Cre mice. Hargreaves data are from 8 sessions from 2 female and 2 male PV-Cre mice. Acetone data are from 8 sessions from 2 female and 2 male PV-Cre mice. **(C)** Behavioral outcomes in the von Frey, Hargreaves, and acetone tests after CNI during sham control. Von Frey data are from 11 sessions from 2 female and 2 male PV-Cre mice. Hargreaves data are from 4 sessions from 2 female and 2 male mice. Acetone data are from 4 sessions from 2 female and 2 male PV-Cre mice. **(D)** Normalized differences between the injured and uninjured hindpaws during CNI baseline, excitation of PV+ neurons in the BLA, and sham control conditions. Von Frey data are from 59 session from 2 female and 2 male PV-Cre mice. Hargreaves data are from 16 session from 2 female and 2 male PV-Cre mice. Acetone data are from 16 session from 2 female and 2 male PV-Cre mice. Triangle symbols denote female animals while square symbols denote male animals. Each symbol represents an individual animal. Error bars indicate 1 S.E.M.

**Figure 3:**
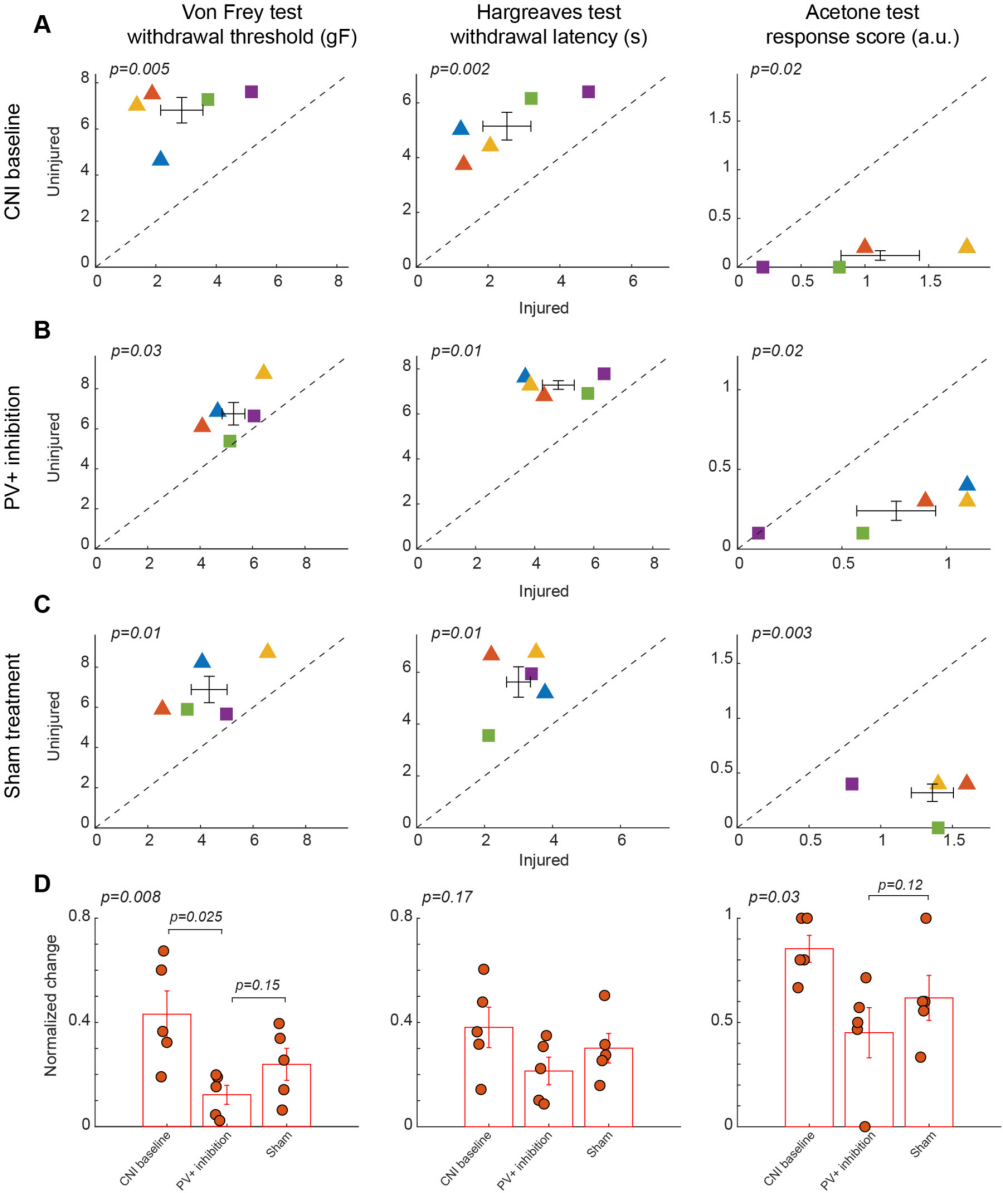
Effects of inhibition of PV+ interneuron in the BLA. **(A)** Behavioral outcomes in the von Frey, Hargreaves, and acetone tests after CNI. Von Frey data are from 17 sessions from 3 female and 2 male PV-Cre mice. Hargreaves data are from 5 sessions from 3 female and 2 male PV-Cre mice. Acetone data are from 5 sessions from 3 female and 2 male PV-Cre mice. **(B)** Behavioral outcomes in the von Frey, Hargreaves, and acetone tests after CNI with inhibition of PV+ neurons in the BLA. Von Frey data are from 40 sessions from 3 female and 2 male PV-Cre mice. Hargreaves data are from 10 sessions from 3 female and 2 male PV-Cre mice. Acetone data are from 10 sessions from 3 female and 2 male PV-Cre mice. **(C)** Behavioral outcomes in the von Frey, Hargreaves, and acetone tests after CNI during sham control. Von Frey data are from 15 sessions from 3 female and 2 male PV-Cre mice. Hargreaves data are from 5 sessions from 3 female and 2 male PV-Cre mice. Acetone data are from 5 sessions from 3 female and 2 male PV-Cre mice. **(D)** Normalized differences between the injured and uninjured hindpaws during CNI baseline, inhibition of PV+ neurons in the BLA, and sham control conditions. Von Frey data are from 72 sessions from 3 female and 2 male PV-Cre mice. Hargreaves data are from 20 sessions from 3 female and 2 male PV-Cre mice. Acetone data are from 20 sessions from 3 female and 2 male PV-Cre mice. Triangle symbols denote female animals while square symbols denote male animals. Each symbol represents an individual animal. Error bars indicate 1 S.E.M.

**Figure 4:**
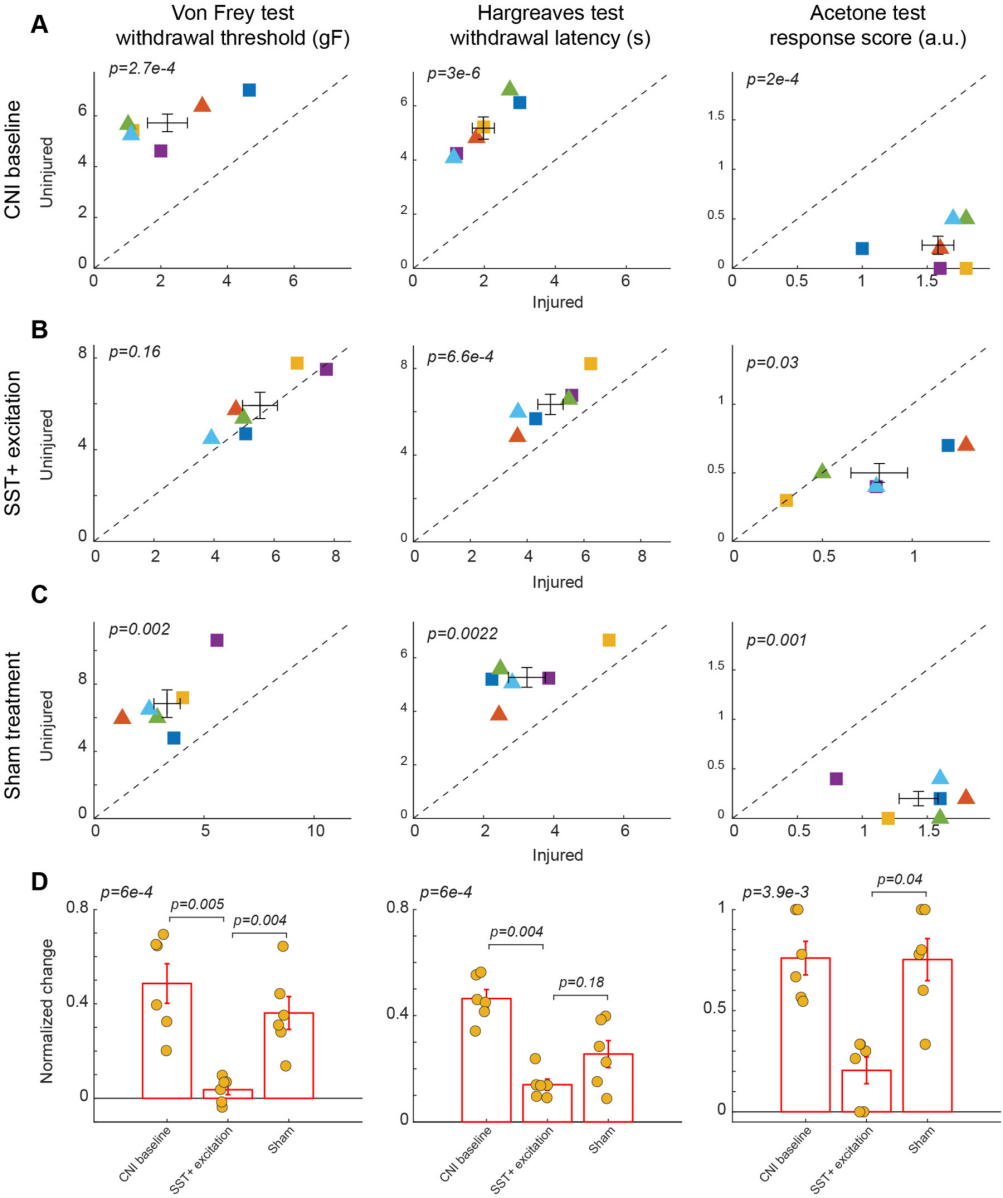
Effects of activation of SST+ interneuron in the BLA. **(A)** Behavioral outcomes in the von Frey, Hargreaves, and acetone tests after CNI. Von Frey data are from 24 sessions from 3 female and 3 male SST-Cre mice. Hargreaves data are from 6 sessions from 3 female and 3 male SST-Cre mice. Acetone data are from 6 sessions from 3 female and 3 male SST-Cre mice. **(B)** Behavioral outcomes in the von Frey, Hargreaves, and acetone tests after CNI with activation of SST+ neurons in the BLA. Von Frey data are from 40 sessions from 3 female and 3 male SST-Cre mice. Hargreaves data are from 12 sessions from 3 female and 3 male SST-Cre mice. Acetone data are from 12 sessions from 3 female and 3 male SST-Cre mice. **(C)** Behavioral outcomes in the von Frey, Hargreaves, and acetone tests after CNI during sham control. Von Frey data are from 18 sessions from 3 female and 3 male SST-Cre mice. Hargreaves data are from 6 sessions from 3 female and 3 male SST-Cre mice. Acetone data are from 6 sessions from 3 female and 3 male SST-Cre mice. **(D)** Normalized differences between the injured and uninjured hindpaws during CNI baseline, excitation of SST+ neurons in the BLA, and sham control conditions. Von Frey data are from 82 sessions from 3 female and 3 male SST-Cre mice. Hargreaves data are from 24 sessions from 3 female and 3 male SST-Cre mice. Acetone data are from 24 sessions from 3 female and 3 male SST-Cre mice. Triangle symbols denote female animals while square symbols denote male animals. Each symbol represents an individual animal in panels **A–C**. Error bars indicate 1 S.E.M.

To assess the behavioral outcome of constriction nerve injury (CNI), we first measured the baseline sensitivity to mechanical (von Frey), thermal (infrared heat), and cold (acetone) stimuli prior to the induction of CNI (Figure 1). Before the injury, we did not find significant differences between the two hindpaws in sensitivity to the mechanical (6.69 ± 0.15 vs. 6.54 ± 0.17, *p* = 0.47, paired Student's *t*-test; Figure 1D), and cold stimulus (0.175 ± 0.039 vs. 0.125 ± 0.038, *p* = 0.27, Wilcoxon Signed Rank test; Figure 1E). However, there was a small but significant difference in the latency in response to thermal stimuli between the two hindpaws (6.74 ± 0.25 vs. 5.86 ± 0.27, *p* = 0.007, paired Student's *t*-test; Figure 1F). We also segregated the baseline data by sex and found that the results of the von Frey tests were consistent between male and female animals (Supplementary Figure S1). After CNI, the mice exhibited hypersensitivity to mechanical and thermal stimulus in the injured hindpaw compared to the uninjured paw (Figures 2–4), confirming the neuropathic pain model.

### Excitation of PV+ interneurons in the BLA alleviates mechanical but not thermal allodynia

Following the CNI, mice developed robust mechanical, thermal, and cold hypersensitivity in the hindpaw on the injured side compared to the uninjured side. For mice with excitatory DREADD receptors expressed in parvalbumin-expressing (PV+) neurons in the basolateral amygdala (BLA), their mechanical paw withdrawal thresholds were significantly reduced for the injured side as compared to the uninjured side (2.07 ± 0.4 vs. 6.56 ± 0.65 g, *p* = 0.003, paired Student's *t*-test, Figure 2A left panel. Supplementary Figure S2 for session-wise data). Similarly, the withdrawal latencies of the paw on the injured side in response to infrared heat (Hargreaves test) were shorter (1.83 ± 0.32 vs. 4.49 ± 0.3 s, *p* = 0.001, paired Student's *t*-test, Figure 2A middle panel), and their acetone-evoked cold responses were enhanced compared to the hindpaw on the uninjured side (1.35 ± 0.21 vs. 0.05 ± 0.05, *p* = 0.005, paired Student's *t*-test) (Figure 2A, right panel). These changes were consistent with persistent allodynia and hyperalgesia induced by CNI (1, 34). Given that these animals showed little difference in these assays between the two paws prior to CNI, the observed hypersensitivity following nerve injury confirmed the replication of a neuropathic pain model upon which we will subsequently test the effects of various chemogenetic manipulations on pain perception.

Chemogenetic activation of PV-expressing GABAergic interneurons in the BLA significantly ameliorated neuropathic pain behaviors induced by CNI in von Frey tests. Following CNO-mediated activation of PV+ neurons, the mice exhibited a marked increase in mechanical withdrawal thresholds for the injured paw, resulting in a comparable threshold to the uninjured paw (8.7 ± 0.51 vs. 7.84 ± 0.47, *p* = 0.16, paired Student's *t*-test, Figure 2B, left panel. Supplemental Figure S2B). As expected, the administration of saline, which served as a sham control for CNO-mediated activation of PV+ neurons in the BLA, did not decrease the difference in von Frey withdrawal threshold between the injured and uninjured hindpaws (5.08 ± 0.23 vs. 7.89 ± 0.38, *p* = 0.005, paired Student's *t*-test, Figure 2C, left panels. Supplementary Figure 2C for session-wise data).

To further quantify the effect of the excitation of PV+ neurons in the BLA, we calculated the normalized difference in all responses between the injured and uninjured paws, which is the difference of the measure between the two paws over the sum of the measure of the two paws (see Methods). A normalized difference of 0 would indicate that the injured and uninjured paws had the same response to the behavioral tests. For the von Frey test, the excitation of BLA PV+ neurons decreased the normalized difference induced by the CNI, and maintained a significant difference with response to saline (*p* = 0.007, *post-hoc* Tukey-Kramer test; *p* = 1.5 × 10^−4^, repeated measures ANOVA test for three conditions, Figure 2D, left panel. Supplementary Figure S2D for session-wise data).

Similarly, heat hypersensitivity was improved by the activation of PV+ neurons in the BLA, with differences in the withdrawal latencies between the two hindpaws vanishing after excitation of BLA PV+ neurons (6.04 ± 0.97 vs. 6.65 ± 1.38, *p* = 0.35, paired Student's *t*-test, Figure 2B, middle panel). However, in the saline control sessions, the difference between the two hindpaws was not statically significant (5.61 ± 1.96 vs. 8.31 ± 1.09, *p* = 0.25, paired Student's *t*-test, Figure 2C, middle panel), probably due to the small number of samples. When looking at the normalized difference between PV+ excitation and saline control sessions for Hargreaves tests, although there is a significant difference among the groups (*p* = 0.038, repeated measures ANOVA test), the normalized difference during BLA PV+ excitation was not significantly different than that in sham control conditions (*p* = 0.40, *post-hoc* Tukey-Kramer test, Figure 2D, middle panel), suggesting that the excitation of PV+ interneurons in the BLA only minimally reduces thermal allodynia.

In the acetone induced cold test, we still observed a significant difference in the response score following the excitation of PV+ neurons in the BLA (0.48 ± 0.08 vs. 0.13 ± 0.05, *p* = 0.012, paired Student's *t*-test, Figure 2B, right panel). In the saline controls, the differences in the response score between the two hindpaws were marginally significant (1.05 ± 0.25 vs. 0.15 ± 0.1, *p* = 0.05, paired Student's *t*-test, Figure 2C, right panel). However, the normalized difference was about the same between the PV+ excitation sessions and the saline control sessions (*p* = 0.29, repeated measures ANOVA test, Figure 2D, right panel), indicating that the excitation of PV+ interneurons in the BLA has no beneficial effects.

Taken together, these results suggest that the activation of PV+ interneurons in the BLA selectively alleviates mechanical allodynia while exerting little effects on heat and cold hypersensitivity.

### Inhibition of PV+ interneurons in the BLA had no effect on neuropathic hypersensitivity

In contrast, chemogenetic inhibition of PV+ interneurons in the BLA did not significantly improve mechanical, thermal, or cold hypersensitivity induced by CNI. In the von Frey tests, paw withdrawal thresholds differed significantly between ipsilateral and contralateral hindpaws following CNI (2.86 ± 0.7 vs. 6.81 ± 0.55 g, *p* = 0.005, paired Student's *t*-test, Figure 3A, left panel; Supplementary Figure S3A for session-wise data) and during the sham control (4.33 ± 0.68 vs. 6.88 ± 0.66, *p* = 0.01, paired Student's *t*-test, Figure 3C, left panel; Supplementary Figure S3C for session-wise data), indicating the injured side remained significantly more sensitive than the contralateral side. However, following CNO-mediated inhibition of PV+ interneurons, mechanical paw withdrawal thresholds retained a significant difference between the injured and uninjured sides (5.28 ± 0.43 vs. 6.75 ± 0.56 g, *p* = 0.03, paired Student's *t*-test, Figure 3B, left panel; Supplementary Figure S3B for session-wise data). We next examined whether the normalized differences between the two hindpaws differed between the PV+ inhibition and sham control conditions. Although there was a decrease in normalized difference following inhibition of BLA PV+ neurons compared to sham control, the difference was not statistically significant (*p* = 0.15, *post-hoc* Tukey-Kramer test, Figure 3D, left panel), suggesting that the inhibition of PV+ neurons in the BLA had little effect on mechanical hypersensitivity.

As with responses indicating mechanical paw withdrawal threshold, although the injured hindpaw exhibited hypersensitivity in Hargreaves test following CNI (2.52 ± 0.67 vs. 5.15 ± 0.51, *p* = 0.002, paired Student's *t*-test, Figure 3A, middle panel) and during sham control (3 ± 0.35 vs. 5.62 ± 0.59, *p* = 0.01, paired Student's *t*-test, Figure 3C, middle panel), the thermal sensitivity of the mice was not restored to near-contralateral latencies following inhibition of PV+ neurons in the BLA (4.8 ± 0.54 vs. 7.3 ± 0.19, *p* = 0.01, paired Student's *t*-test, Figure 3B, middle panel). Following calculation of normalized differences, we found that responses to PV+ inhibition and saline administration did not show a statistically significant difference (*p* = 0.17, repeated measures ANOVA test, Figure 3D, middle panel), suggesting that inhibition of PV+ neurons in the BLA has no effect on heat allodynia.

Similarly, in the acetone induced cold test, although there was a significant difference between two hindpaws after CNI (1.12 ± 0.31 vs. 0.12 ± 0.05, *p* = 0.02, paired Student's *t*-test, Figure 3A, right panel), we still observed significant differences in the response following inhibition of BLA PV+ neurons (0.76 ± 0.19 vs. 0.24 ± 0.06, *p* = 0.02, paired Student's *t*-test, Figure 3B, right panel). As expected, saline control results also showed significant difference between hindpaws (1.36 ± 0.15 vs. 0.32 ± 0.08, *p* = 0.003, paired Student's *t*-test, Figure 3C, right panel). Normalized difference analysis confirmed that there is no significant difference in response between PV+ inhibition and saline control (*p* = 0.12, *post-hoc* Tukey-Kramer test, Figure 3D, right panel). These findings suggest that inhibition of PV+ interneuron activity is insufficient to normalize sensory thresholds. The persistence of mechanical, thermal, and cold hypersensitivity under PV+ inhibition underscores the specificity of BLA PV+ neuron activation in counteracting allodynia.

### Excitation of SST+ interneurons in the BLA reduces mechanical and cold, but not heat hypersensitivity

After characterizing the effect of manipulation of PV+ neurons in the BLA on pain-related behavior, we next examined the role of somatostatin (SST)-expressing neurons in the BLA in pain perception. Activation of SST+ neurons in the BLA diminished mechanical allodynia induced by CNI (2.2 ± 0.6 vs. 5.72 ± 0.35, *p* = 2.7 × 10^−4^, paired Student's *t*-test, Figure 4A, left panel; Supplementary Figure S4A for session-wise data). Following CNO-mediated activation of BLA SST+ interneurons, mice showed a significant increase in mechanical paw-withdrawal threshold for the injured paw, reaching values nearly equivalent to uninjured paw responses (5.53 ± 0.58 vs. 5.93 ± 0.57, *p* = 0.16, paired Student's *t*-test, Figure 4B, left panel; Supplementary Figure S4B for session-wise data). Consistent with results in PV+ manipulation experiments, sham control with saline administration did not decrease the difference in response between injured and uninjured paws (3.32 ± 0.6 vs. 6.84 ± 0.82, *p* = 0.002, paired Student's *t*-test, Figure 4C, left panel; Supplementary Figure S4C for session-wise data).

We further calculated the normalized difference between the injured and uninjured paws for the von Frey test. Repeated measures ANOVA test confirmed a statistically significant difference among the three groups (*p* = 6 × 10^−4^). Activation of BLA SST+ interneurons significantly reduced the normalized difference induced by CNI compared to sham treatment (*p* = 0.004, *post-hoc* Tukey-Kramer test, Figure 4D, left panel; Supplementary Figure S4D for session-wise data).

Activation of BLA SST+ neurons failed to improve heat hypersensitivity as thermal withdrawal latencies differed significantly during CNI baseline (1.97 ± 0.31 vs. 5.18 ± 0.41, *p* = 3 × 10^−6^, paired Student's *t*-test, Figure 4A, middle panel), SST+ activation (4.82 ± 0.44 vs. 6.34 ± 0.47, *p* = 6.6 × 10^−4^, paired Student's *t*-test, Figure 4B, middle panel), and sham control (3.24 ± 0.52 vs. 5.27 ± 0.37, *p* = 2.2 × 10^−3^, paired Student's *t*-test, Figure 4C, middle panel). There also was a non-significant difference in the normalized differences between SST+ activation and saline sham control (*p* = 0.18, *post-hoc* Tukey-Kramer test, Figure 4D, middle panel). This suggests that SST+ activation does not reduce thermal allodynia.

Unlike the manipulation of PV+ neurons in the BLA, activation of SST+ neurons in the BLA appeared to improve cold-induced allodynia, as indicated by a reduction in acetone-evoked responses between uninjured and injured paws (0.82 ± 0.16 vs. 0.5 ± 0.07, *p* = 0.03, paired Student's *t*-test, Figure 4B, right panel) as compared to in Saline control sessions (1.43 ± 0.15 vs. 0.2 ± 0.07, *p* = 0.001, paired Student's *t*-test, Figure 4C, right panel). This was further confirmed by their normalized differences (*p* = 0.04, *post-hoc* Tukey-Kramer test, Figure 4D, right panel.) These results highlight that SST+ interneuron excitation not only normalizes mechanical hypersensitivity, but also extends its analgesic effects to cold hypersensitivity.

### Inhibition of SST+ interneurons improves mechanical and heat, but not cold, hypersensitivity

**Figure 5:**
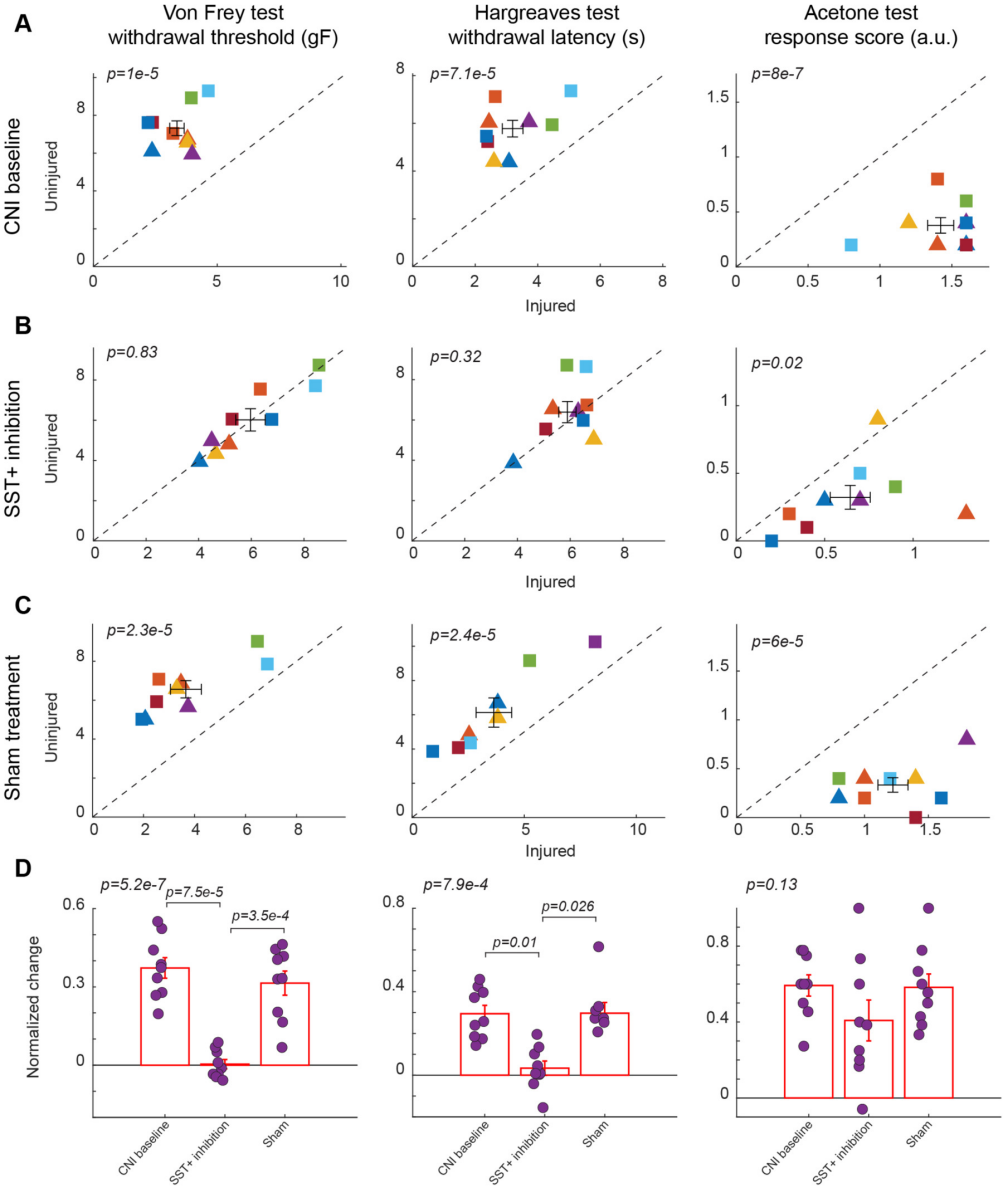
Effects of inhibition of SST+ interneuron in the BLA. **(A)** Behavioral outcomes in the von Frey, Hargreaves, and acetone tests after CNI. Von Frey data are from 35 sessions from 4 female and 5 male SST-Cre mice. Hargreaves data are from 9 sessions from 4 female and 5 male SST-Cre mice. Acetone data are from 9 sessions from 4 female and 5 male SST-Cre mice. **(B)** Behavioral outcomes in the von Frey, Hargreaves, and acetone tests after CNI with inhibition of SST+ neurons in the BLA. Von Frey data are from 72 sessions from 4 female and 5 male SST-Cre mice. Hargreaves data are from 19 sessions from 4 female and 5 male SST-Cre mice. Acetone data are from 18 sessions from 4 female and 5 male SST-Cre mice. **(C)** Behavioral outcomes in the von Frey, Hargreaves, and acetone tests after CNI during sham control. Von Frey data are from 27 sessions from 4 female and 5 male SST-Cre mice. Hargreaves data are from 8 sessions from 3 female and 5 male SST-Cre mice. Acetone data are from 9 sessions from 4 female and 5 male SST-Cre mice. **(D)** Normalized differences between the injured and uninjured hindpaws during CNI baseline, inhibition of SST+ neurons in the BLA, and sham control conditions. Von Frey data are from 134 sessions from 4 female and 5 male SST-Cre mice. Hargreaves data are from 36 sessions from 4 female and 5 male SST-Cre mice. Acetone data are from 36 sessions from 4 female and 5 male SST-Cre mice. Triangle symbols denote female animals while square symbols denote male animals. Each symbol represents an individual animal in panels **A–C**. Error bars indicate 1 S.E.M.

We found that chemogenetic inhibition of SST+ neurons in the BLA also conferred a relief from mechanical and heat hypersensitivity. In response to CNO-mediated inhibition of SST+ neurons, there was a significant increase in mechanical paw withdrawal thresholds for the injured side to uninjured levels (5.97 ± 0.56 vs. 6.02 ± 0.27, *p* = 0.83, paired Student's *t*-test, Figure 5B, left panel; Supplementary Figure S5B for session-wise data), while the significant difference between injured and uninjured hindpaws was observed in both CNI baseline (3.37 ± 0.29 vs. 7.32 ± 0.39, *p* = 1 × 10^−5^, paired Student's *t*-test, Figure 5A, left panel; Supplementary Figure S5A for session-wise data) and saline control sessions (3.66 ± 0.6 vs. 6.56 ± 0.44, *p* = 2.3 × 10^−5^, paired Student's *t*-test, Figure 5C, left panel; Supplementary Figure S5C for session-wise data). After calculation of normalized difference, there was a significant difference between BLA SST+ inhibition condition and saline control (*p* = 3.5 × 10^−4^, *post-hoc* Tukey-Kramer test, Figure 5D, left panel; Supplementary Figure S5D for session-wise data).

Unlike BLA SST+ activation, BLA SST+ inhibition resulted in improved thermal hypersensitivity, with injured paw response reaching contralateral levels (5. 89 ± 0.33 vs. 6.39 ± 0.52, *p* = 0.32, paired Student's *t*-test, Figure 5B, middle panel). Responses to saline control mirrored CNI baseline response, maintaining a significant difference between injured and uninjured hindpaws (*p* = 7.1 × 10^−5^, paired Student's *t*-test, Figure 5A, middle panel, and *p* = 2.4 × 10^−5^, paired Student's *t*-test, Figure 5C, middle panel). There was also a significant difference between SST+ inhibition and saline administration when comparing the normalized difference (*p* = 0.026, *post-hoc* Tukey-Kramer test, Figure 5D, middle panel).

However, the inhibition of BLA SST^+^ neurons did not alter the injured paw's response to acetone-induced cold, as the injured paw consistently exhibited hypersensitivity compared to the uninjured paw across CNI baseline (1.42 ± 0.09 vs. 0.38 ± 0.07, *p* = 8 × 10^−7^, paired Student's *t*-test, Figure 5A, right panel), SST^+^ inhibition (0.64 ± 0.11 vs. 0.32 ± 0.09, *p* = 0.02, paired Student's *t*-test, Figure 5B, right panel), and sham treatment conditions (1.22 ± 0.12 vs. 0.33 ± 0.07, *p* = 6 × 10^−5^, paired Student's *t*-test, Figure 5C, right panel). There were also no statistically significant differences between CNI, SST+ inhibition, and saline administration when examining the normalized differences (*p* = 0.13, repeated measures ANOVA test, Figure 5D, right panel).

## Discussion

Our results demonstrate that both PV^+^ and SST^+^ interneurons in the BLA contribute to the regulation of nociceptive processing, but they do so in modality-specific ways. Excitation of PV^+^ neurons in the BLA robustly alleviated mechanical allodynia. This is in line with the results of a previous study, where excitation of PV^+^ neurons in the spinal cord in mice with nerve injury attenuated their mechanical hypersensitivity, whereas transiently silencing the spinal cord PV+ neurons in naive mice resulted in mechanical allodynia (37). This improvement is likely because, in the BLA, perisomatic inhibition by PV^+^ neurons induces a strong and immediate decrease in the firing rate of excitatory neurons (27, 28). Upon nerve injury, the reduced expression of PV in PV^+^ neurons disrupted firing patterns, thereby decreasing the overall inhibition exerted by PV^+^ neurons (38). Therefore, chemogenetic excitation of PV^+^ neurons restored inhibition exerted by PV^+^ neurons in the BLA and reinstated mechanical sensitivity. We failed to find PV^+^ inhibition to normalize hypersensitivity for mechanical, cold, or heat stimuli. This is consistent with previous studies suggesting that inhibition by PV^+^ neurons is already impaired following nerve injury (38, 39). For instance, Dang et al. (39) found a reduction in PV+ neurons in the BLA and their activation in rats 8 weeks following a nerve injury caused by partial sciatic nerve ligation. Our results provide new evidence suggesting that neural plasticity involving PV^+^ neurons in the BLA plays an important role in mediating pain behavior following nerve injury.

By contrast, both excitation and inhibition of SST^+^ neurons in the BLA improved mechanical allodynia, suggesting a more nuanced role for these neurons in pain processing. The observation that both activation and inhibition of BLA SST^+^ interneurons produced similar pain-relieving effects in response to mechanical stimulation raises important questions regarding their circuit-level function. One potential explanation for this bidirectional effect is that SST^+^ neurons participate in homeostatic or compensatory mechanisms following nerve injury. Similar feedback loops have been observed in cortical circuits, where inhibitory plasticity adjusts to maintain network stability after injury or pathological hyperactivity (40, 41). In the BLA, a comparable mechanism may exist, wherein any perturbation of SST^+^ neuron function—whether by increasing or decreasing their activity—disrupts maladaptive pain circuitry and restores nociceptive thresholds. This could be mediated by reciprocal connections between SST^+^ and other inhibitory interneurons, such as PV^+^ or vasoactive intestinal peptide (VIP^+^) neurons (20, 42).

Interestingly, excitation of SST^+^ neurons in the BLA improved animals' cold hypersensitivity but not their thermal hypersensitivity, while inhibition of SST^+^ neurons in the BLA does the opposite. This indicates that SST^+^ interneurons may play different roles in circuits processing thermal and cold information. This aligns with findings in other brain regions where SST^+^ interneurons can either suppress excitatory output or disinhibit downstream targets depending on the state of the network (43, 44). Our findings also raise a critical question whether pain processing across different modalities (mechanical, thermal, and cold) engages distinct neural circuits involving the same interneuron populations within the BLA. The fact that manipulation of BLA SST^+^ neurons affects pain behavior in all three modalities, while PV^+^ manipulation was only effective for mechanical and thermal hypersensitivities, suggests a potential link between nociceptive pathways and interneuron function. Cold hypersensitivity is often mediated by transient receptor potential (TRP) channels, including TRPM8 and TRPA1, which may preferentially interact with SST^+^-regulated circuits (45). In contrast, mechanical and thermal pain pathways, which rely more heavily on TRPV1 and ASIC channels, may be more tightly coupled to PV^+^ interneuron function (46). Future studies employing cell-type-specific manipulations combined with nociceptive receptor tracing could further elucidate these relationships.

By dissecting the roles of PV^+^ and SST^+^ interneurons in the BLA, our study provides new insights into the circuit mechanisms underlying chronic pain. While PV^+^ excitation strongly modulates mechanical and thermal pain, SST^+^ neurons exhibit a more complex, bidirectional influence on nociceptive thresholds, particularly in cold allodynia. These findings suggest that distinct interneuron populations engage different nociceptive pathways and feedback loops, opening new possibilities for precision-targeted therapies for chronic pain. Beyond local inhibitory interneurons, our findings also implicate BLA-PFC and BLA-PAG circuits in chronic pain modulation. Previous studies have shown that inhibition of BLA projections to the PFC alleviates pain in mice with nerve injury (47), reinforcing the idea that amygdala-prefrontal pathways contribute to pain perception. Given that PV^+^ and SST^+^ neurons regulate BLA output, they may indirectly shape these descending pain-modulatory circuits, either amplifying or suppressing pain perception at a systems level (11, 12).

There are several limitations of the current study. First, we did not confirm selective expression of DREADD receptors in the BLA PV+ or SST+ neurons for all experimental animals, raising the possibility of off-target effects. However, Cre-dependent expression of transgenes via AAV delivery in PV-Cre and SST-Cre mice has been widely validated in numerous previous studies (48, 49). The distribution of putative SST+ neurons in the BLA observed in our study is consistent with earlier reports (Figure 1C) (50). Moreover, in the two experimental SST-Cre mice which we randomly selected to perform histological analysis, DREADD receptor expression appeared to be correctly localized within the BLA, validating our AAV delivery techniques (Figure 1C). Importantly, the behavioral data from these two mice are consistent with those of the remaining cohort, suggesting that DREADD receptor expression was predominantly on-target as intended. Another limitation of the study is the relatively small number of animals used. Although the study included two genotypes and two manipulation conditions, each cohort consisted of only 4–9 mice. Future studies with larger sample sizes will be important to validate and extend the findings reported here. Finally, although we used each animal as its own control by comparing data from injured and uninjured paws, unilateral neuropathic injury may influence sensory processing on the contralateral side. Future studies performing similar manipulations in mice without nerve injury could provide valuable insights into the extent to which BLA PV+ or SST+ neuron activity alone is sufficient to drive the observed behavioral phenotypes.

Nevertheless, the results of the present study suggest several important directions for future research. First, high-resolution viral tracing and single-cell transcriptomics could help distinguish SST^+^ and PV^+^ subpopulations within the BLA, revealing whether distinct interneuron subtypes preferentially regulate specific pain modalities (24, 51). Second, targeted pharmacological strategies that enhance SST-peptide signaling or boost PV^+^ interneuron function could offer novel therapeutic avenues for chronic pain management (40, 44). Finally, investigating how neuromodulatory inputs (e.g., noradrenergic, cholinergic, etc.) interact with inhibitory microcircuits may clarify how these interneurons shape pain processing in a state-dependent manner (52–55). Understanding how these microcircuits involving PV^+^ and SST^+^ interneurons and their interaction with broader pain networks will be crucial for developing effective neuromodulation interventions for chronic pain conditions.

## Data Availability

The raw data supporting the conclusions of this article will be made available by the authors, without undue reservation.
