# Supplementary material for: Bidirectional modulation of somatostatin-expressing interneurons in the basolateral amygdala reduces neuropathic pain perception in mice
Source: Front Pain Res (Lausanne). 2025 Aug 13;6:1602036. doi: 10.3389/fpain.2025.1602036 (PMC12380630; doi:10.3389/fpain.2025.1602036)
Supplement: Supplementary file 1 [file Image1.pdf]

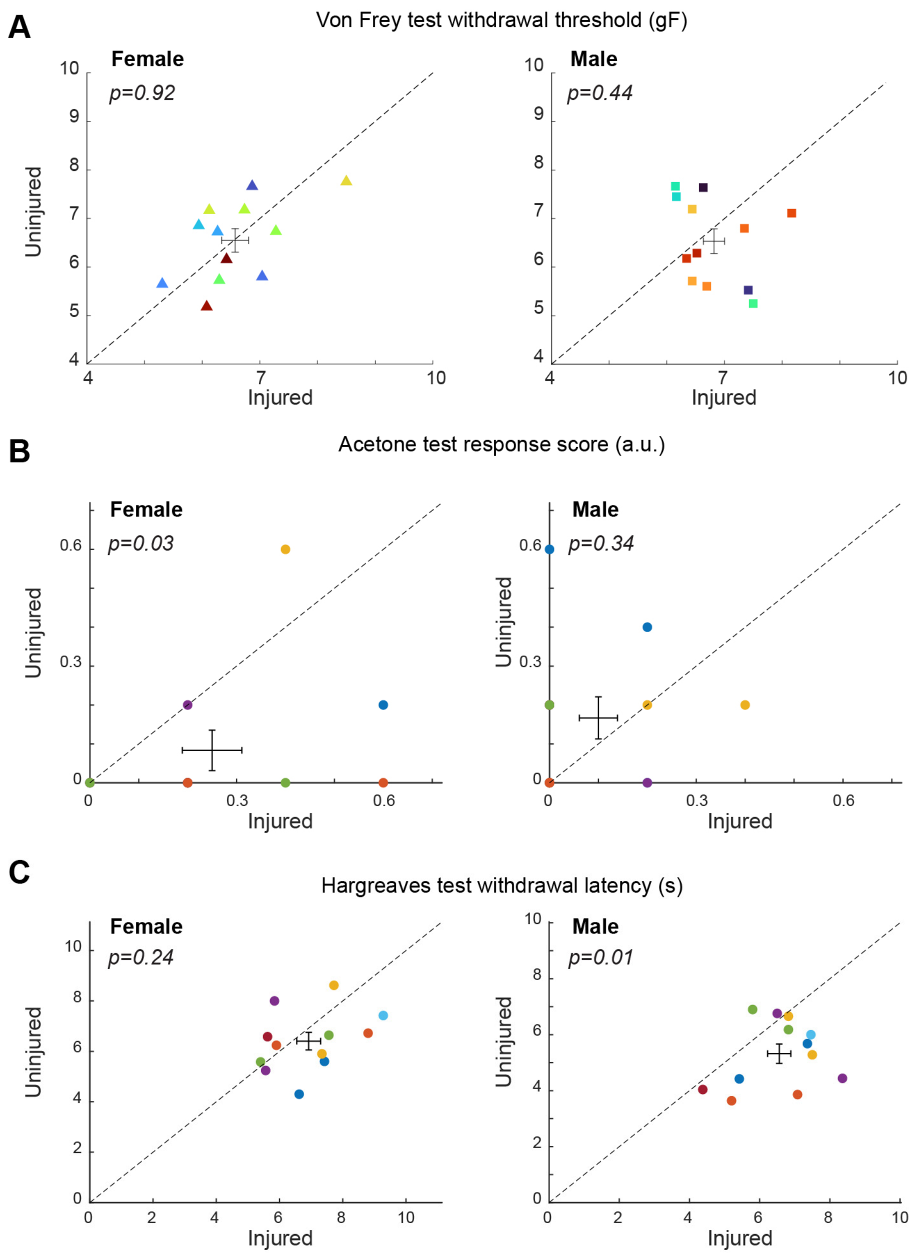

Supplemental Figure 1: Pre-CNI behavioral results segregated by sex. A) *The von Frey test results for female and male animals. Data from 52 sessions from 12 female mice and 54 sessions from 12 male mice.* ***B)*** *The Hargreaves test results for female and male animals. Data from 12 sessions from 12 female mice and 12 sessions from 12 male mice.* ***C)*** *The acetone test results for female and male animals. Data from 12 sessions from 12 female mice and 12 sessions from 12 male mice. Each circle indicates a session, and each color represents an individual animal. Error bars indicate 1 S.E.M.*

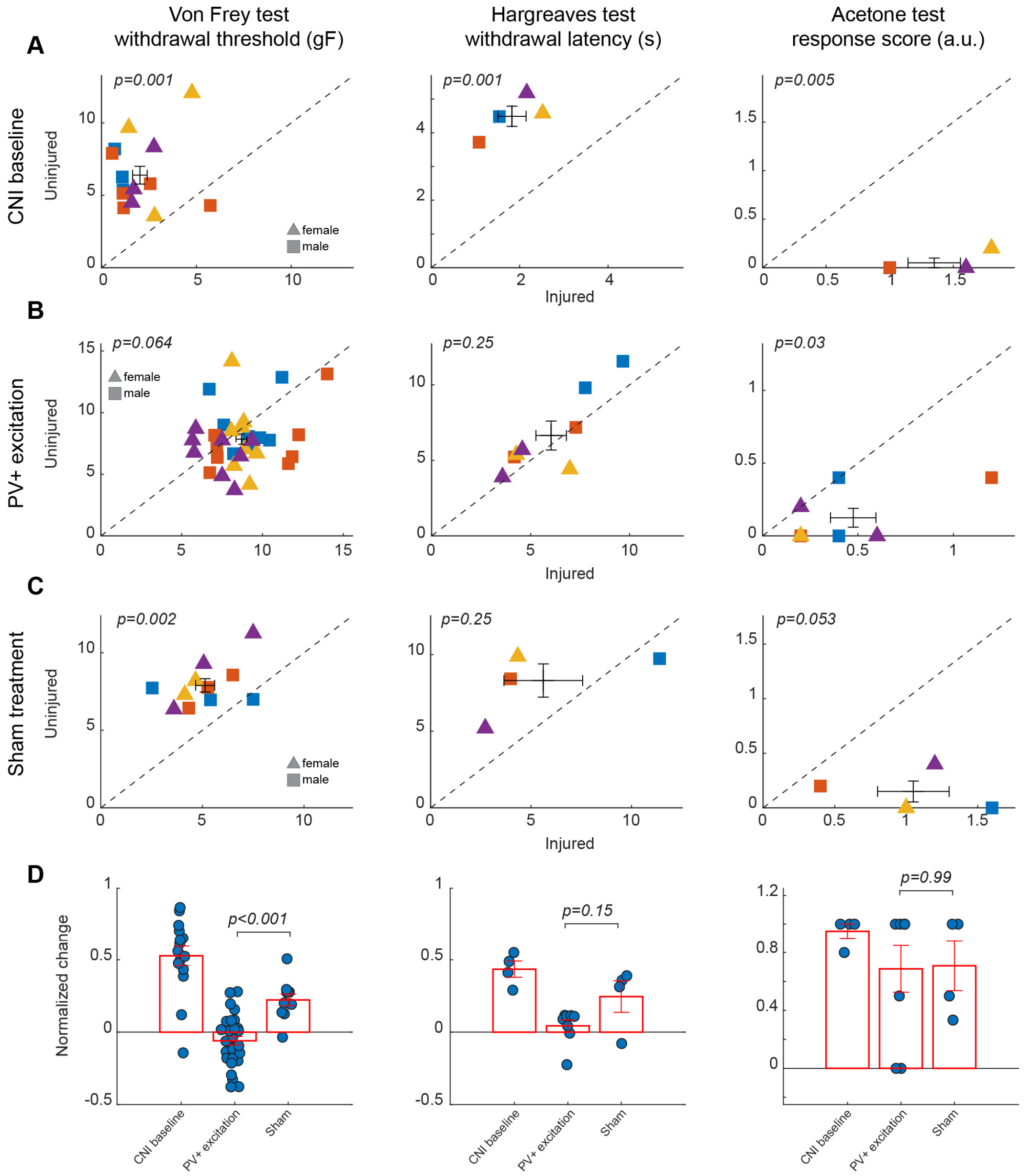

Supplemental Figure 2: Effects of activation of PV+ interneuron in the BLA plotted on a session-wise basis. A) *Behavioral outcomes in the von Frey, Hargreaves, and acetone tests after CNI. Von Frey data are from 15 sessions from 2 female and 2 male PV-Cre mice. Hargreaves data are from 4 sessions from 2 female and 2 male PV-Cre mice. Acetone data are from 4 sessions from 2 female and 2 male PV-Cre mice.* ***B)*** *Behavioral outcomes in the von Frey, Hargreaves, and acetone tests after CNI with activation of PV+ neurons in the BLA. Von Frey data are from 33 sessions from 2 female and 2 male PV-Cre mice. Hargreaves data are from 8 sessions from 2 female and 2 male PV-Cre mice. Acetone data are from 8 sessions from 2 female and 2 male PV-Cre mice.* ***C)*** *Behavioral outcomes in the von Frey, Hargreaves, and acetone tests after CNI during sham control. Von Frey data are from 11 sessions from 2 female and 2 male PV-Cre mice. Hargreaves data are from 4 sessions from 2 female and 2 male mice. Acetone data are from 4 sessions from 2 female and 2 male PV-Cre mice.* ***D)*** *Normalized differences between the injured and uninjured hindpaws during CNI baseline, excitation of PV+ neurons in the BLA, and sham control conditions. Von Frey data are from 59 session from 2 female and 2 male PV-Cre mice. Hargreaves data are from 16 session from 2 female and 2 male PV-Cre mice. Acetone data are from 16 session from 2 female and 2 male PV-Cre mice. Triangle symbols denote female animals while square symbols denote male animals. Each symbol indicates a session, and each color represents an individual animal in panels A-C. Error bars indicate 1 S.E.M.*

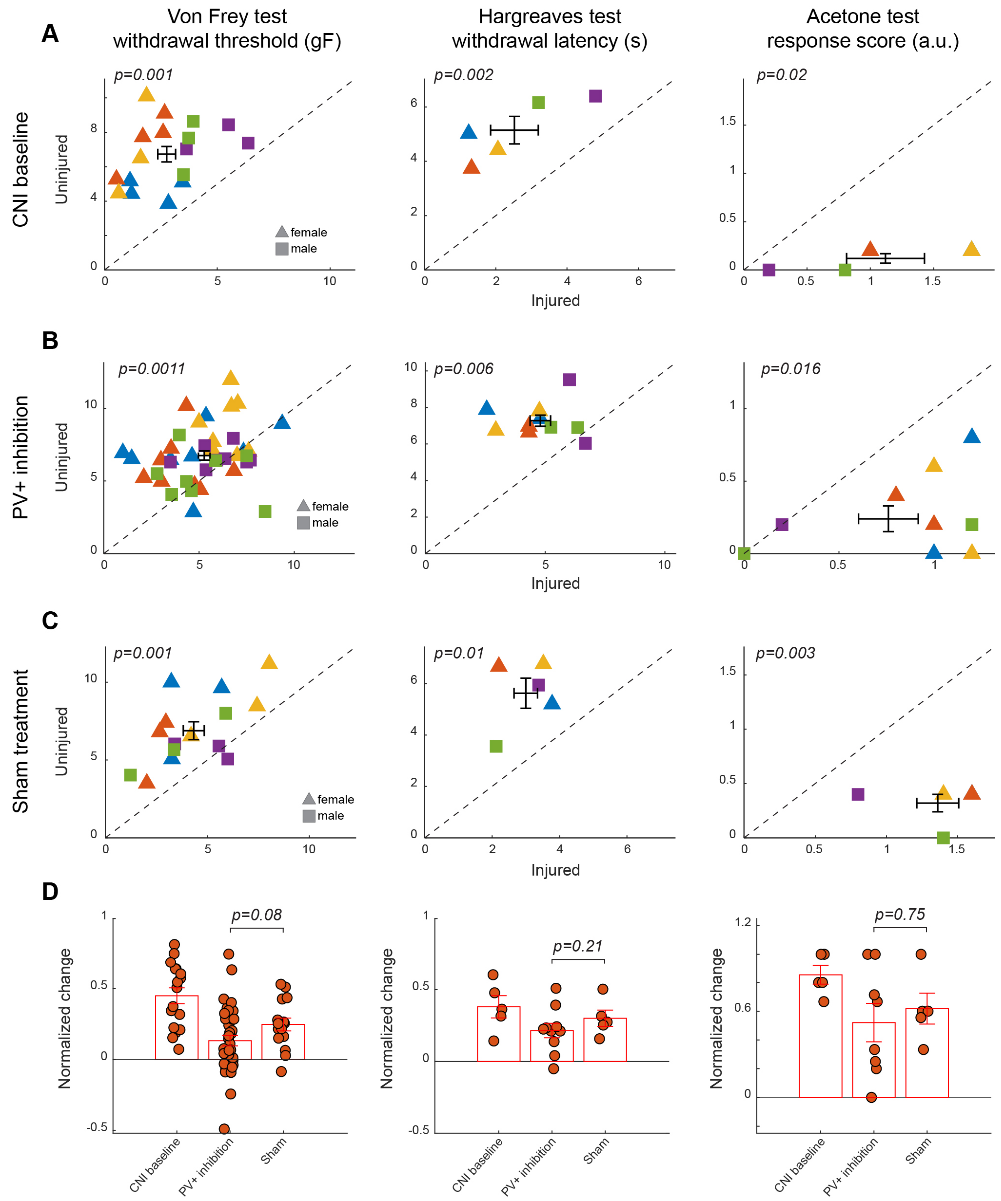

Supplemental Figure 3: Effects of inhibition of PV+ interneuron in the BLA plotted on a session-wise basis. A) *Behavioral outcomes in the von Frey, Hargreaves, and acetone tests after CNI. Von Frey data are from 17 sessions from 3 female and 2 male PV-Cre mice. Hargreaves data are from 5 sessions from 3 female and 2 male PV-Cre mice. Acetone data are from 5 sessions from 3 female and 2 male PV-Cre mice.* ***B)*** *Behavioral outcomes in the von Frey, Hargreaves, and acetone tests after CNI with inhibition of PV+ neurons in the BLA. Von Frey data are from 40 sessions from 3 female and 2 male PV-Cre mice. Hargreaves data are from 10 sessions from 3 female and 2 male PV-Cre mice. Acetone data are from 10 sessions from 3 female and 2 male PV-Cre mice.* ***C)*** *Behavioral outcomes in the von Frey, Hargreaves, and acetone tests after CNI during sham control. Von Frey data are from 15 sessions from 3 female and 2 male PV-Cre mice. Hargreaves data are from 5 sessions from 3 female and 2 male PV-Cre mice. Acetone data are from 5 sessions from 3 female and 2 male PV-Cre mice.* ***D)*** *Normalized differences between the injured and uninjured hindpaws during CNI baseline, inhibition of PV+ neurons in the BLA, and sham control conditions. Von Frey data are from 72 sessions from 3 female and 2 male PV-Cre mice. Hargreaves data are from 20 sessions from 3 female and 2 male PV-Cre mice. Acetone data are from 20 sessions from 3 female and 2 male PV-Cre mice. Triangle symbols denote female animals while square symbols denote male animals. Each symbol indicates a session, and each color represents an individual animal in panels A-C. Error bars indicate 1 S.E.M.*

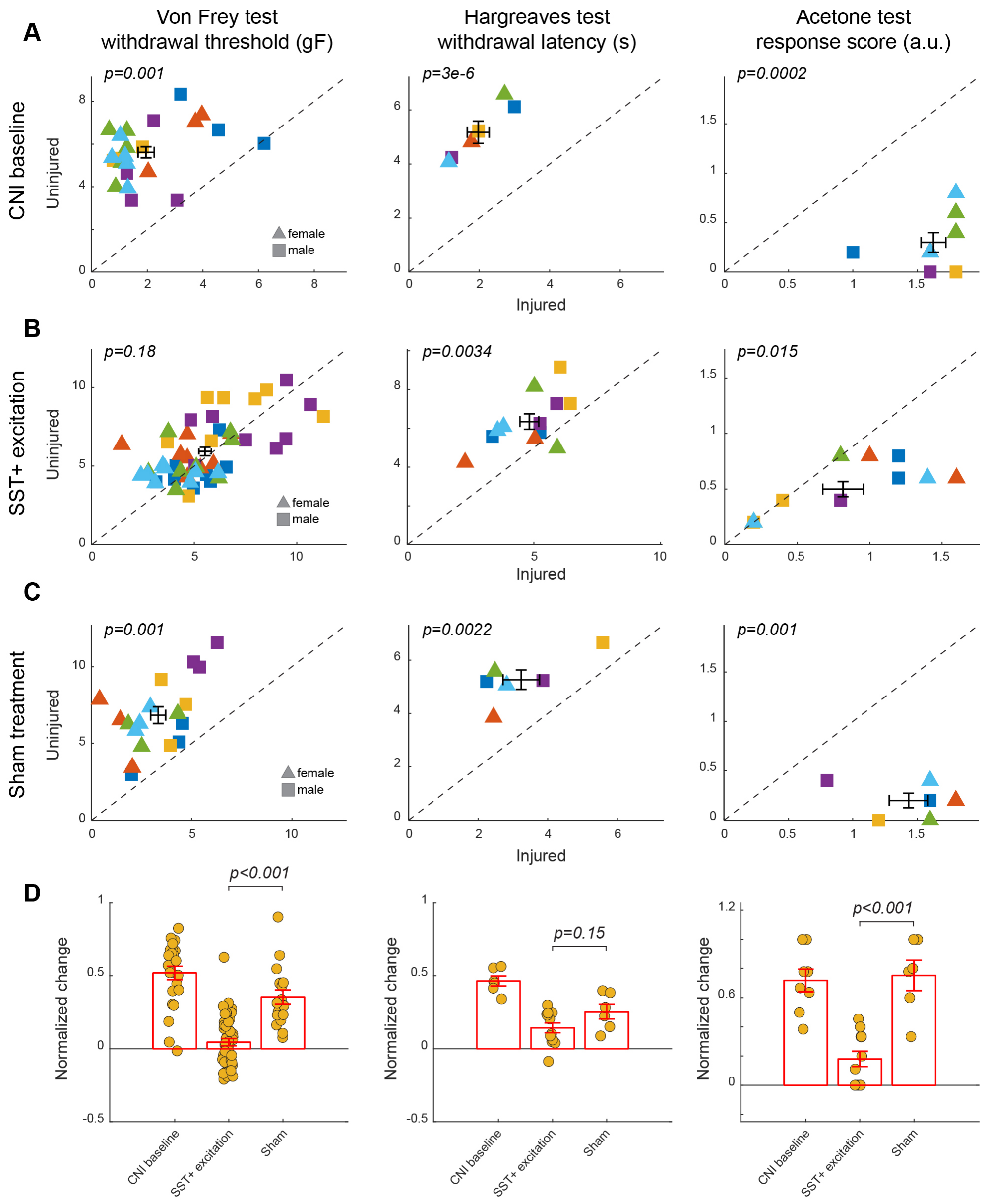

Supplemental Figure 4. Effects of activation of SST+ interneuron in the BLA plotted on a session-wise basis. A) *Behavioral outcomes in the von Frey, Hargreaves, and acetone tests after CNI. Von Frey data are from 24 sessions from 3 female and 3 male SST-Cre mice. Hargreaves data are from 6 sessions from 3 female and 3 male SST-Cre mice. Acetone data are from 6 sessions from 3 female and 3 male SST-Cre mice.* ***B)*** *Behavioral outcomes in the von Frey, Hargreaves, and acetone tests after CNI with activation of SST+ neurons in the BLA. Von Frey data are from 40 sessions from 3 female and 3 male SST-Cre mice. Hargreaves data are from 12sessions from 3 female and 3 male SST-Cre mice. Acetone data are from 12 sessions from 3 female and 3 male SST-Cre mice.* ***C)*** *Behavioral outcomes in the von Frey, Hargreaves, and acetone tests after CNI during sham control. Von Frey data are from 18 sessions from 3 female and 3 male SST-Cre mice. Hargreaves data are from 6 sessions from 3 female and 3 male SST-Cre mice. Acetone data are from 6 sessions from 3 female and 3 male SST-Cre mice.* ***D)*** *Normalized differences between the injured and uninjured hindpaws during CNI baseline, excitation of SST+ neurons in the BLA, and sham control conditions. Von Frey data are from 82 sessions from 3 female and 3 male SST-Cre mice. Hargreaves data are from 24 sessions from 3 female and 3 male SST-Cre mice. Acetone data are from 24 sessions from 3 female and 3 male SST-Cre mice. Triangle symbols denote female animals while square symbols denote male animals. Each symbol indicates a session, and each color represents an individual animal in panels A-C. Error bars indicate 1 S.E.M.*

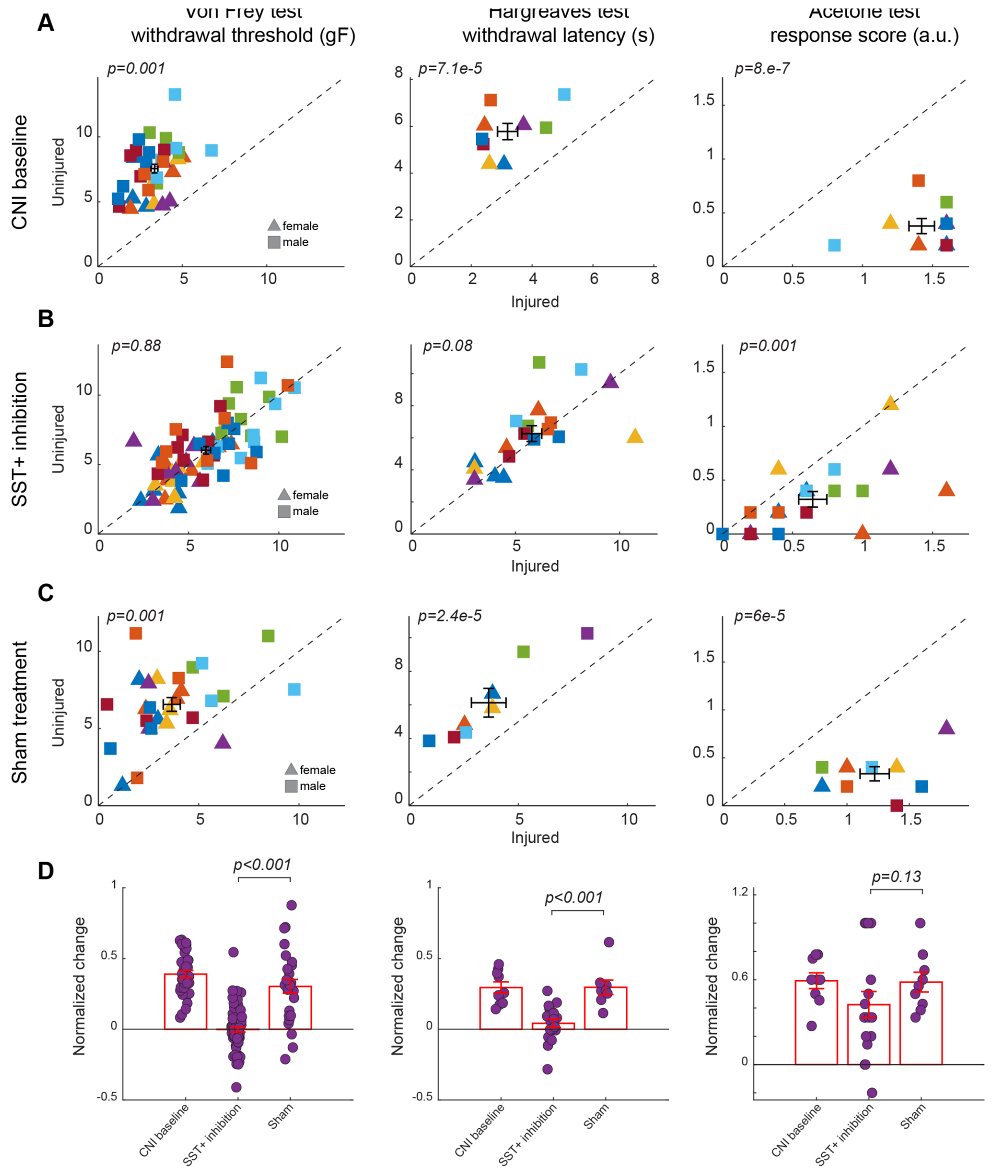

Supplemental Figure 5: Effects of inhibition of SST+ interneuron in the BLA plotted on a session-wise basis. A) *Behavioral outcomes in the von Frey, Hargreaves, and acetone tests after CNI. Von Frey data are from 35 sessions from 4 female and 5 male SST-Cre mice. Hargreaves data are from 9 sessions from 4 female and 5 male SST-Cre mice. Acetone data are from 9 sessions from 4 female and 5 male SST-Cre mice.* ***B)*** *Behavioral outcomes in the von Frey, Hargreaves, and acetone tests after CNI with inhibition of SST+ neurons in the BLA. Von Frey data are from 72 sessions from 4 female and 5 male SST-Cre mice. Hargreaves data are from 19 sessions from 4 female and 5 male SST-Cre mice. Acetone data are from 18 sessions from 4 female and 5 male SST-Cre mice.* ***C)*** *Behavioral outcomes in the von Frey, Hargreaves, and acetone tests after CNI during sham control. Von Frey data are from 27 sessions from 4 female and 5 male SST-Cre mice. Hargreaves data are from 8 sessions from 3 female and 5 male SST-Cre mice. Acetone data are from 9 sessions from 4 female and 5 male SST-Cre mice.* ***D)*** *Normalized differences between the injured and uninjured hindpaws during CNI baseline, inhibition of SST+ neurons in the BLA, and sham control conditions. Von Frey data are from 134 sessions from 4 female and 5 male SST-Cre mice. Hargreaves data are from 36 sessions from 4 female and 5 male SST-Cre mice. Acetone data are from 36 sessions from 4 female and 5 male SST-Cre mice. Triangle symbols denote female animals while square symbols denote male animals. Each symbol indicates a session, and each color represents an individual animal in panels A-C. Error bars indicate 1 S.E.M.*
